# KinMutRF: a random forest classifier of sequence variants in the human protein kinase superfamily

**DOI:** 10.1186/s12864-016-2723-1

**Published:** 2016-06-23

**Authors:** Tirso Pons, Miguel Vazquez, María Luisa Matey-Hernandez, Søren Brunak, Alfonso Valencia, Jose MG Izarzugaza

**Affiliations:** Structural Biology and BioComputing Programme, Spanish National Cancer Research Centre (CNIO), Melchor Fernández Almagro, 3, 28029 Madrid, Spain; Center for Biological Sequence Analysis (CBS), Systems Biology Department, Technical University of Denmark (DTU), Kemitorvet, Building 208, 2800 Kgs., Lyngby, Denmark; Novo Nordisk Foundation Center for Protein Research, Faculty of Health Sciences, University of Copenhagen, Blegdamsvej 3A, 2200 Copenhagen, Denmark

**Keywords:** Protein kinases, Variant prioritization, Pathogenicity prediction, Functional impact, X-linked agammaglobulinemia

## Abstract

**Background:**

The association between aberrant signal processing by protein kinases and human diseases such as cancer was established long time ago. However, understanding the link between sequence variants in the protein kinase superfamily and the mechanistic complex traits at the molecular level remains challenging: cells tolerate most genomic alterations and only a minor fraction disrupt molecular function sufficiently and drive disease.

**Results:**

KinMutRF is a novel random-forest method to automatically identify pathogenic variants in human kinases. Twenty six decision trees implemented as a random forest ponder a battery of features that characterize the variants: a) at the gene level, including membership to a Kinbase group and Gene Ontology terms; b) at the PFAM domain level; and c) at the residue level, the types of amino acids involved, changes in biochemical properties, functional annotations from UniProt, Phospho.ELM and FireDB. KinMutRF identifies disease-associated variants satisfactorily (Acc: 0.88, Prec:0.82, Rec:0.75, F-score:0.78, MCC:0.68) when trained and cross-validated with the 3689 human kinase variants from UniProt that have been annotated as neutral or pathogenic. All unclassified variants were excluded from the training set. Furthermore, KinMutRF is discussed with respect to two independent kinase-specific sets of mutations no included in the training and testing, Kin-Driver (643 variants) and Pon-BTK (1495 variants). Moreover, we provide predictions for the 848 protein kinase variants in UniProt that remained unclassified.

A public implementation of KinMutRF, including documentation and examples, is available online (http://kinmut2.bioinfo.cnio.es). The source code for local installation is released under a GPL version 3 license, and can be downloaded from https://github.com/Rbbt-Workflows/KinMut2.

**Conclusions:**

KinMutRF is capable of classifying kinase variation with good performance. Predictions by KinMutRF compare favorably in a benchmark with other state-of-the-art methods (i.e. SIFT, Polyphen-2, MutationAssesor, MutationTaster, LRT, CADD, FATHMM, and VEST). Kinase-specific features rank as the most elucidatory in terms of information gain and are likely the improvement in prediction performance. This advocates for the development of family-specific classifiers able to exploit the discriminatory power of features unique to individual protein families.

**Electronic supplementary material:**

The online version of this article (doi:10.1186/s12864-016-2723-1) contains supplementary material, which is available to authorized users.

## Background

Only a minor fraction of the large number of variants discovered with current high-throughput next generation sequencing (NGS) methodologies are causally implicated in disease onset [1–6]. The correct identification of the causative variants remains a challenging effort [7]. For a few examples there is sufficient experimental information associating variants and human maladies, and for an even smaller number of cases the underlying biochemical mechanism is known. However, for the vast majority of the sequence variants identified, ~100,000 disease-associated variants, the functional information is missing [8]. The experimental characterization and functional annotation of those novel variants would require humongous resources. Nevertheless, this problem is very amenable to computational approaches [6]. Different methods to predict the probability of a variant being causaly implicated in a disease have been proposed during the last decade. A brief description of the most popular methods, along with relevant URLs and references, are listed in Additional file 1: Table S1. A first group of methods applied deterministic rules to a reduced number of protein features to identify damaging mutations. For example, the widely cited methods SIFT [9] and MutationAssessor [10], MutPred [11], FATHMM [12], Panther [13] and PROVEAN [14] rely on different interpretations of signatures of evolutionary constraint to assess the pathogenicity of variants. A second group of methods (e.g. PMUT [15], SNAP [16], PolyPhen-2 [17], NetDiseaseSNP [18], LS-SNP [19], PhD-SNP [20], MutationTaster [21], VEST [22], SNPs&GO [23], SNPs3D [24], MuD [25], CanPredict [26], CADD [27], PON-P2 [28] and nsSNPAnalyzer [29]) rely on advanced automatic machine learning approaches that integrate prior knowledge in the form of both sequence-based and structure-based features, under the assumption that pathogenic variants will disrupt normal protein function and structural stability. After a training process where the system is presented a set of previously characterized damaging and neutral variants, new variants can be classified based on the knowledge acquired. Each method implements a different machine learning approach: neural networks [15, 16, 18], Bayesian methods [17, 21], support vector machines [19, 20, 23, 24, 27] or random forests [22, 25, 26, 28, 29]. Recently, some meta-predictor have been published, for instance, Meta-SNP [30] combines four of the most widely employed computational methods for prioritising missense single nucleotide variations, both Condel [31] and PON-P [32] integrate five classifiers, and PredictSNP [33] incorporates eight. Moreover, the SPRING [34] method is based on six functional effect scores calculated by existing methods (SIFT, Polyphen2, LRT, MutationTaster, GERP and PhyloP) and five association scores derived from a variety of genomic data sources (Gene Ontology, protein protein interactions, protein sequences, protein domain annotations and gene pathway annotations). Concomitantly, each predictor implements a distinctive set of features with a different scope and applicability. Some predictors are generally applicable to any protein, while a recent group of methods include properties that focus on a characteristic subset of variants (eg. Cancer variants predicted by CanPredict [26], CanDrA [35] and CHASM [36]) or a protein family of interest under the assumption that family-specific features bring discriminative information that justifies the development of specialized methods. An interesting example of the latter are protein kinases [5, 37–40]. The protein kinase superfamily is very amenable to this approach. Protein kinases play a central role in the cell and consequently they have been studied in detail. As a consequence, a broad number of variants in members of the protein kinase superfamily have been reported in the literature in relation to disease [41], including some types of cancer [42]. In previous publications, we demonstrated the preferential distribution of both germline and somatic variants [43, 44] around regions of functional and structural relevance and how this information can be used to develop a computational method [37] to predict the impact of variants on the function of protein kinases. The combination of the predictions from the classifier with annotations extracted from the literature and other sources, facilitates the mechanistical interpretation of the consequences of the variants [45].

Here, we introduce KinMutRF as a random forest-based classifier to predict the pathogenicity of novel variants. Although the core functionality builds up on our previous work [37], in this new implementation we redefine the sequence-derived features, using optimized ways to extract the signals encoded at the protein, domain and residue levels. To demonstrate the improved prediction capabilities of the KinMutRF, approach we benchmark our random forest classifier with other state-of-the-art prediction methods and we discuss the benefits and pitfalls of the development of a family-specific predictor in the light of our findings.

## Methods

### Training datasets

Variants affecting members of the protein kinase superfamily were downloaded from the UniProt/Swiss-Prot variant pages (release 2014_08 of 03-Sept-2014) [46], which compile variants in UniProtKB. The training datasets used in this work have been included with the Supplementary Materials.

### Statistics to evaluate prediction performance

Accordig to best practices in the field [46–48], perfomances was assesed in terms of Accuracy, Precision, Recall, F-score and Mathew’s correlation coefficient (MCC).

$$ Accuracy=\frac{TP+TN}{TP+TN+FP+FN} $$

$$ Precision=\frac{TP}{TP+FP} $$

$$ Recall=\frac{TP}{TP+FN} $$

$$ F- score=\frac{2}{Precisio{n}^{-1}+ Recal{l}^{-1}} $$

$$ MCC=\frac{TP\times TN-FP\times FN}{\sqrt{\left(TP+FP\right)\left(TP+FN\right)\;\left(TN+FP\right)\left(TN+FN\right)}} $$

Where:

TP: True positives, correctly predicted pathogenic variants; FP: False positives, neutral variants predicted as disease prone; TN: True negatives, correctly predicted neutral variants; and FN: False negatives, pathogenic variants predicted as neutral.

### Description of the classification features

**Fig. 1:**
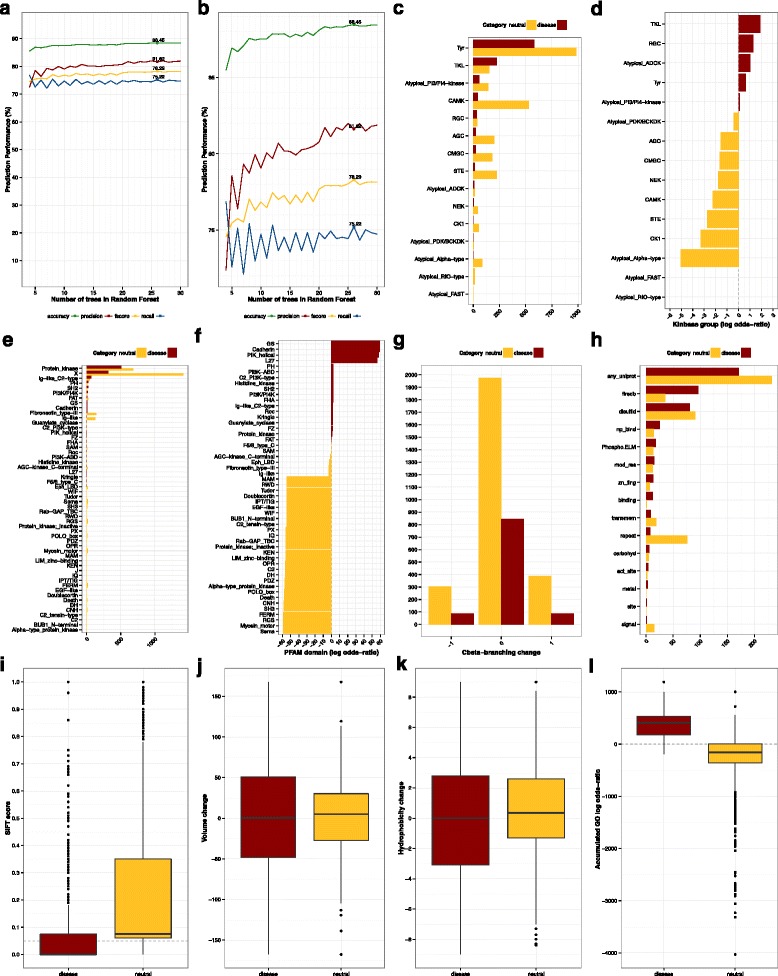
Performance and classification features. **a** Performance of the classifier respect to the number of trees in the random forest; **b** *idem,* close-up on the region around the performance values; **c** Number of variants in each kinase group; **d** log odds-ratio of the number of variants in each kinase group; **e** Number of variants in each kinase domain; **f** log odds-ratio of the number of variants in each kinase domain; **g** changes in Cbeta-branching caused by pathogenic and neutral variants; **h** number of pathogenic and neutral variants affecting catalytic sites as defined by UniProt, FireDB and Phospho.ELM. **i** Distribution of SIFT scores; **j** Changes in volume caused by disease-associated and neutral variants; **k** Changes in hydrophobicity caused by disease-associated and neutral variants; **l** Accumulated Gene Ontology (GO) log odds-ratio. Note that, where relevant, disease-associated variants were represented in dark red whereas ochre was used for their neutral counterparts

Variants were characterized with a battery of 25 features at the protein, domain and residue level (see details below). The distribution of variants in the training sets respect the classification features can be found in Fig. 1 (panels from c to l). Classification features were computed as follows:

#### Membership to kinase groups

We used the taxonomy proposed by Manning [49] implemented in UniProt to classify the protein kinases superfamily. This taxonomy considers three levels of abstraction: subfamilies, families and groups. The level of protein kinase groups are stablished according to sequence similarity, the presence of accessory domains, and by considering the different modes of regulation. For a detailed description of protein kinase groups in KinBase and the abbreviations used in this work, see reference [50] and the supplementary materials. A total of 15 protein kinase groups were considered in this analysis (Fig. 1, panels c and d) and the log odds ratio of their contribution to disease was calculated according to the following formula:

$$ kinase\; group=lo{g}_2\frac{\left( disease\;var.\kern0.24em  in\; kinase\; group+\upxi \right)/ disease\;var.}{\left( neutral\;var.\kern0.24em  in\; kinase\; group+\upxi \right)/ neutral\;var.} $$

Where “disease var.” and “neutral var.” refer to the total number of variants in UniProt classified as disase or neutral, respectively. The terms “disease var. in kinase group” and “neutral var. in kinase group” are the number of variants in a specific kinase group for each category. Note that a pseudo count of ξ = 10^-20^ is considered to resolve kinase groups with no neutral variants.

#### Gene ontology terms (sumGOlor)

Gene Ontology (GO) annotations were used as a proxy for the functional relevance of protein kinases. Starting from the terms that annotate each kinase in UniProt the three subontologies (i. e. molecular function, biological process and cellular compartment) were followed to their roots to consider all parent nodes. The probabilities of observing each of these GO terms together with neutral and disease variants were compared with log-odds ratio (Fig. 1, panel l). Protein kinase are characterised by the sum of the individual contributions of their GO terms.

$$ sumGOlor={\displaystyle \sum_{i=1}^nlo{g}_2}\frac{\left( disease\;var.\kern0.24em  annotated\; with\kern0.24em GOi+\upxi \right)/ disease\;var.}{\left( neutral\;var.\kern0.24em  annotated\; with\kern0.24em GOi+\upxi \right)/ neutral\;var.} $$

Where “disease var.” and “neutral var.” refer to the total number of variants in UniProt classified as disase or neutral, respectively. The terms “disease var. annotated with GOi” and “neutral var. annotated with GOi” are the number of variants annoatated with a particular gene ontology term for each category, disease-associated or neutral. Note that a pseudo count of ξ = 10^-20^ is considered to resolve cases where no neutral variants where annotated with GO_i_.

#### PFAM domains

For each of the 80 different domains defined by UniProt as found in the protein kinase superfamily, a log-odds ratio (details in Fig. 1, panels e and f) of the frequency with which they harbour disease and neutral variants has been computed according to the following formula:

$$ sumPFAMlor={\displaystyle \sum_{i=1}^nlo{g}_2}\frac{\left( disease\;var.\; in\; PFAMi+\upxi \right)/ disease\;var.}{\left( neutral\;var.\; in\; PFAMi+\upxi \right)\;/ neutral\;var.} $$

Where “disease var.” and “neutral var.” refer to the total number of variants in UniProt classified as disase or neutral, respectively. The terms “disease var. in PFAMi” and “neutral var. in PFAMi” are the number of variants in a specific kinase PFAM domain for each category. Note that a pseudo count of ξ = 10^-20^ is considered to resolve cases where no neutral variants where annotated with PFAMi.

#### Amino acid and their biochemical properties

The physic-chemical properties of the amino acids involved in variation often determine the propensity to disease. Our prediction features consider the native amino acid, the newly observed one, and the derived changes in some crucial biochemical properties. These include changes volume, Kyte-Doolittle hydrophobicity, C_beta_ branching and formal charge represented as differences in the nominal values (Fig. 1, panels g, j and k).

#### Residue conservation: SIFT

Variants are described with the precomputed SIFT [51] scores downloaded from dbNSFP [52] as a proxy for amino acid conservation at the position of interest (Fig. 1, panel i). Conservation within a set of related sequences has traditionally been the strongest and most widely implemented features for the classification of variants.

#### Functional annotations in UniProt, FireDB and Phospho.ELM

The activity of protein kinases is affected by the alteration of functionally relevant residues involved, for example, in catalysis or phosphorilation. In the implementation of KinMutRF, residue annotations in UniProt [53] define functionally relevant amino acids. The residue annoations include the following categories: active sites (act_site), general (binding) or specialised binding (carbohyd, metal, np_bind), disulfid bonding, experimentally modified residues (mod_res), repeat regions (repeat), signal peptides (signal), transmembrane regions (transmem) and zinc fingers (zn_fing), among others broadly defined sites. An additional categories (any_uniprot) account for the residues being annotated with at least one of the previous categories. Similarly, phosphorilation sites from Phospho.ELM [54] and for the prediction of the catalytic and ligand-binding sites according to FireDB [55] are included (Fig. 1, panel h).

## Results and discussion

### Construction of the training datasets

Variants affecting members of the protein kinase superfamily were extracted from the UniProt/Swiss-Prot variant pages [46], the compilation of variation available in UniProtKB. Every variant in this set is given a classification as neutral or pathogenic. In the few cases were the same variant was described by several instances, a single record was considered, selecting a pathogenic instance if ambiguous. Note that no additional reclassification attending to disease types or information from other sources was applied. After the filtering process, 1021 unique variants in 84 protein kinases form the disease dataset and 2668 variants in 450 proteins conform its neutral counterpart. In total, there were variants described and classified for 459 out of the 507 protein kinases described in UniProt, and 75 kinases span both categories of variants. The disease and neutral variant sets were used for training and evaluation of the machine learning classifier. The 848 variants affecting 299 kinases that are listed as unclassified in UniProt were left out from this analysis.

The training of the random forest-based classification kernel of KinMutRF followed a 10-fold cross-validation approach. As suggested by the best practices in the field [16, 46], the 459 protein kinases for which classified variation data exists were distributed randomly in 10 different bins. All variants corresponding to an individual protein were assigned to the same bin. We incorporated this rule to avoid overestimating the performance of the classification; the contrary would constitute a circularity type 2 bias [47, 56]. This bias might originate from similarities at the protein level (i.e. different variants from the same protein) between the training and evaluation sets. To ensure reproducibility of our results and to facilitate of other methods to be developed in the future, these training bins have been included with the Supplementary Materials (Additional file 2: Supplementary File S1). Then, each bin was iteratively used as evaluation set whereas the remaining nine were used as training instances. Results are accumulated until all bins had been used in the evaluation step. Following current standard practice in the field [47–49], we assessed the performance of the clasiffier with five different statistics: accuracy, precision, recall, f-score and Mathew’s correlation coefficient (MCC) according to the formulas described in Methods.

### Optimization of the prediction method

A machine learning classifier was trained to predict the pathogenicity of variants affecting the human kinome. In particular, a Random Forest kernel was selected after exploration of the many methods implemented in the Weka (v.3.6.11) package. To optimise the parametrization of the random forest classifier, we explored an increasing number of decision trees, ranging from 4 to 30 elements. Our results (Fig. 1, panels a and b) show that all performance statistics reach a steady plateau after an expected initial overhead and suggest that prediciton performance is not afffected by moderate alterations in the size of the forest. Subsequent analyses implement a configuration with 26 trees given the slightly better f-score in average in our preliminary analyses.

### Evaluation of classification performance in the training set

In a previous section we described the construction of the training datasets and how these were used in 10-fold crossvalidation experiment to assess the prediction capabilities of the KinMutRF classifier according to five common statistics. Accuracy accounts for the fraction of variants correctly predicted in function of the total number of variants. Due to the innate inbalance in the constitution of the datasets, with 1021 neutral variants and 2668 disease-associated variants respectively, a naïve classifier predicting every variant as the majority class would achieve a basal 72.32 % accuracy. Consequently, the evaluation of the classification should refer to the prediction of the positive class. In the case of a predictor of pathogenicity, this corresponds to the pathogenic mutations. Precision accounts for the proportion of correctly predicted disease-associated variants with respect to all the variants predicted as positive by the classifier. Recall, often referred as sensitivity, accounts for the proportion of correctly predicted disease-associated variants respect to all positive variants present in the dataset. These two statistics combine into a single one, the f-score, which is convenient for evaluation purposes. Finally, we considered the Mathew’s correlation coefficient (MCC) accounts for the performance of both the disease and the neutral prediction. Despite accuracy, this statistic is robust even in cases with dispair class sizes. KinMutRF yields accurate results when both classes are considered (accuracy: 88.45 %, MCC: 0.68). Performance is also satisfactory when only the pathogenic set is considered. KinMutRF achieves a precision of 81.62 % and a recall of 75.22 %, that combined produce an f-score of 78.29 %. The implementation of KinMutRF overcomes our previous KinMut results implementing a support vector machine (SVM) kernel and a different set of prediction features [37, 51] (Acc: 83.29 %, Prec: 60.03 %, Recall: 75.17 %, f-score: 66.7 % and MCC: 0.6). The improvement is particularly significant in terms of precision, the ability to predict correctly in the pathogenic variants, while a similar recall is maintained.

### Most relevant features for classification

The contribution of individual features for the classification of the classes was assesed using the InfoGainAttributeEval module in Weka (v.3.6.11). Features are ranked according to the information gain resulting from the inclusion of individual features. The ranking of the classification features of KinMutRF is summarised in Table 1. One would expect that a family-specific predictor would benefit from the use of the information encoded by features that pertain only to the family of interest. Our ranking of features follows this intuition as the highest information gain (0.491) corresponds to the implementation of Gene Ontology terms that describe the function of each protein kinase and the fequency with which it has been reported in relation with disease and neutral variants (sumGOlor). This observation is coherent with Fig. 1 (panel l), where a clear separation between the accummulated GO log odds ratio of the two classes of variations (disease-associated and neutral). The evolutionary conservation of the residues, measured with SIFT, follows in the ranking. with an information gain of 0.179. In spite of not being a kinase-specific feature, this observation is coherent with the widespread use of SIFT as part of a full body of other classifiers and with the observations in Fig. 1 (panel i). Third and fourth position in this ranking are also occupied by kinase-specific features, namely the membership to a kinase group and the relevance of the kinase domains, produce information gains of 0.120 and 0.112 respectively. It is clear from the observaton of Fig. 1 (panels c, d, e and f) that there is a preferential distribution of disease-associated mutations respect to certain protein kinases and domains. One could argue that the inclusion of features that rely on existing knowledge (e.g. protein and domain specific features) might inherently bias the classification of variants. Albeit partially true from a benchmark perspective, the ability to derive correct predictions from related proteins is the ultimate goal of family-specific methods as the one under consideration here. A different reasoning is that genetic aberrations affecting uncharted regions of the variation-space – i.e. less characterised protein kinases – might result difficult to characterise as predictions would be hindered by lack of data, or on a worst case scenario by the strong contribution of the few exisiting examples. We expect that the wealth of data coming from current sequencing efforts would quickly bridge this knowledge gap and that all elements of the human kinome would present a comparable amount of information. This is also true for the development of family-specific methods outside the protein kinase superfamily, currently limited by lack of sufficient variation information. The ranking of features is continued by other commonly used features. However, their contribution to the information gain is an order of magnitude smaller. These include recurrently implemented by methods that focus on alteration of protein stability (Additional file 1: Supplementary Table S1) such as the nature of the wild-type (0.044) and mutant (0.037) amino acids or the associated change in hydrophobicity (0.037). Last in the ranking appear features that assess the relevance of the residue in terms of catalysis and phosphorylation propensity. Their position in the ranking might be determined by their limited abundance. Nevertheless, these observations are coherent with previous observations that determined that disease-associated variants, independently of their somatic or germline character, did not allocated necessarily on catalytic sites but on the close proximity of these, under the hypothesis that the structural neighbourhood of the functional sites is also determinant for correct protein function [43, 44, 57].

**Table 1:** Relevance of prediction features ranked according to the information gain with respect to the class

Rank	Gain	Feature	Rank	Gain	Feature
1	0.4914	Gene Ontology	14	4.79e-3	Binding (UniProt)
2	0.1787	SIFT	15	4.43e-3	Np_bind (UniProt)
3	0.1197	Kinase group	16	3.38e-3	Repeat (UniProt)
4	0.1121	PFAM domain	17	2.47e-3	Phospho.ELM
5	0.0438	Wild type amino ac.	18	2.37e-3	Zn finger (UniProt)
6	0.0373	Hydrophobicity	19	1.82e-3	Modified res. (UniProt)
7	0.0368	Alternative amino ac.	20	1.51e-3	Metal binding (UniProt)
8	0.0353	Volume change	21	9.4e-4	Signal peptide (UniProt)
9	0.0239	FireDB residue	22	7.71e-4	Active site (UniProt)
10	8.94e-3	Any uniprot	23	6.86e-4	Carbohyd (UniProt)
11	7.70e-3	Formal charge	24	5.02e-4	Site (UniProt)
12	6.80e-3	Cbeta Branching	25	5.33e-5	Transmembrane (UniProt)
13	6.02e-3	Disulfid (UniProt)			

Ranking calculated with the InfoGainAttributeEval function in Weka. Features that are specifically related to the protein kinase superfamily rank among the most informative ones

### Benchmark of the classifier respect to other methods

The capability of KinMutRF to correctly identify pathogenic variants was benchmarked to that of another eight state-of-the-art approaches (Table 2). Evaluation was studied according to the five performance measures described in Methods, KinMutRF yields very satisfactory predictions when the other methods are interrogated about the pathogenicity of the 3689 kinase variants for which UniProt provides a characterization. In fact, our methodology achieves the best accuracy (0.88) and precision (0.82) among the evaluated methods, indicative that the prediction of both neutral and pathogenic mutations is sufficiently reliable. This observation is supported by a Matthew’s correlation coefficient (MCC) of 0.68, comparable to that achieved by the the best in this category, VEST [22]. Our f-score (0.78) is also comparable with the one achieved by VEST, that compensated the lack precison with increased recall. The difference in prediction performance might be bigger in practical terms, as the results of KinMutRF competitors correspond to an optimistic interpretation that might be boosted by a circularity type 1 bias [56]; the set used in the benchmark might include variants already presented to the classifiers during their own training phase [52]. This effect was taxatively avoided in the evaluation of KinMutRF.

**Table 2:** Benchmark of KinMutRF respect to other methods

Method	Accuracy	Precision	Recall	F-score	MCC
MutationTaster	0.56	0.38	**0.96**	0.55	0.36
SIFT	0.68	0.45	0.81	0.58	0.39
Polyphen2:HDIV	0.66	0.44	0.90	0.59	0.42
LRT	0.65	0.45	0.87	0.59	0.39
MutationAssessor	0.76	0.55	0.66	0.60	0.43
CADD	0.76	0.54	0.77	0.64	0.48
Polyphen2:HVAR	0.64	0.53	0.85	0.65	0.50
FATHMM	0.82	0.69	0.63	0.66	0.54
VEST	0.87	0.74	0.82	**0.78**	**0.69**
KinMutRF	**0.88**	**0.82**	0.75	**0.78**	0.68

Prediction performance in a 10-fold cross-validation experiment on the 3689 kinase variants for which UniProt provides a characterization of pathogenicity. In bold, the best score for each performance measure

### Comparison to Kin-Driver manually curated kinase variants

To understand the prediction performance of KinMutRF beyond the training datasets, we evaluated the agreement with an independent source, Kin-Driver [58]. The resource present two quantitative adjantages: First, it includes variants that have not been presented to KinMutRF during its training phase. Second, variants are manually classified according to their consequence on protein activity into activating and deactivating, which allows further understanding of the strengths and weakenesses of our model. KinMutRF correctly predicted 65 out of the 159 (40.88 %) pathogenic variants included in Kin-Driver that were not included in the set used for training our predictor. The drop in performance might be explained by the nature of the consequence of the variants. The random forest correctly identified 21 out of 34 (61.76 %) loss-of-function variants whereas only 44 out of the 125 (35.20 %) gain-of-function variants were classified correctly. This analysis is coherent with previous observations [54, 57] that advocate for the further development of methods to predict the consequences of activating variants as most of the methodologies focus on the disruption of protein function.

### Assessment of KinMutRF with Bruton agammaglobulinaemia tyrosine kinase (BTK) variants

We detailed the KinMutRF prediction results on a well-studied tyrosine kinase domain and compare the predictions with those obtained by PON-BTK [59], a kinase-specific pathogenicity predictor. A total of 158 disease-related variants in 91 residues from the Bruton agammaglobulinaemia tyrosine kinase domain (BTK_HUMAN:402-655) are documented in BTKbase version 8.53. These are freely available at http://structure.bmc.lu.se/idbase/BTKbase/. The predictions by both KinMutRF and PON-BTK for the 1495 possible nonsynonymous variants in the BTK protein kinase domain are summarized in Table 3. KinMutRF prediction results for the BTK are provided in Additional file 3: Supplementary File S3. Data in Table 3 reveals a significant agreement in the prediction of pathogenic variants (967 variants) between KinMutRF and PON-BTK. The disagreement in the prediction of pathogenic variants between these methods is very low; only 36 variants predicted as pathogenic by PON-BTK were predicted as neutral by KinMutRF. Väliaho and colleagues [59] described PON-BTK predictions for two variants: one false negative (p.M587L) and one false positive (p.L460F). The p.L460F variant is predicted as neutral by the two methods, PON-BTK and KinMutRF, while p.M587L is predicted as pathogenic only by KinMutRF. Remarkably, neighbor residues to p.L460F, V458 and T474 are ANP ligand-binding according to FireDB (54), and G462 accomodates two X-linked agammaglobulinemia variants (G462D (VAR_008316) and G462V (VAR_008317)). On the other hand, 4 out of 8 additional methods predicts p.L460F as pathogenic variant. These observations indicates that in certain cases with not conclusive results, Web-Lab experiments should be done. The KinMutRF prediction for p.M587L supported by annotations extracted with the Structure-PPi module [60]: 1) neighbor residues E589 and S592 accommodates cancer-related variants (E589A in malignant melanoma, and S592Y in ovary carcinoma and malignant melanoma); and 2) six residues in the close vicinity contains variants associated to X-linked agammaglobulinemia (OMIM: 300755) (C502F (VAR_006245), C502W (VAR_006246), F583S (VAR_008327), E589D (VAR_008328), E589G (VAR_006265), E589K (VAR_008965), S592P (VAR_006267), V626G (VAR_008333), M630I (VAR_006274), M630K (VAR_006275), and M630T (VAR_008334)). Altogether, these evidences suggested a key role for this BTK region in human diseases.

**Table 3:** Summary of the KinMutRF and PON-BTK prediction results

	Pathogenic			Neutral		
	Prediction	Overlap	Diff.	Prediction	Overlap	Diff.
KinMutRF	1285 (85.9 %)	967		210 (14.1 %)	174	
PON-BTK	1003 (67.1 %)		36	492 (32.9 %)		318

Prediction: indicates the total number of BTK variants predicted as pathogenic and neutral. Numbers in parenthesis represent the percentage from a maximum of 1495 possible nonsynonymous variants. Overlap: total number of BTK variants predicted as pathogenic and neutral by KinMutRF and PON-BTK. Diff.: total number of BTK variants with different predictions by KinMutRF and PON-BTK

### Predicting the pathogenicity of unclassfied variants, recorded in UniProtKB/Swiss-Prot

**Fig. 2:**
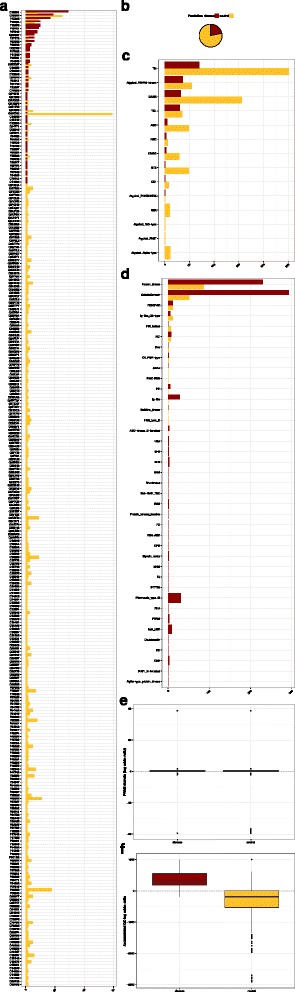
Prediction of pathogenicity for variants uncharacterised in UniProt. **a** Distribution of predictions of pathogenicity in the different protein kinases; **b** Fraction of predictions as disease-associated and neutral; **c** Distribution of predictions of pathogenicity in the different groups in the taxonomy of protein kinases; **d** Distribution of predictions of pathogenicity respect to PFAM domains; **e** Distribution of the PFAM domain log odds-ratios for neutral and disease-associated variants; **f** Distribution of the accummulated Gene Ontology log odds-ratios (sumGOlor) for neutral and disease-associated variants

In a previous section we discussed the preparation of a training set from the variation in UniProtKB/Swiss-Prot variant pages. In this process, we excluded 848 variants in 299 kinases for which a classification of “Disease” and/or “Polymorphism” was not available. We propose that KinMutRF can bridge this gap in knowledge and suggest whether these are most likely pathogenic or neutral. KinMutRF predicted 185 (21.81 %) of these variants as pathogenic (Fig. 2, panel b). The full list of predictions, as well as the prediction features that originated them, can be found with the Supplementary Materials (Additional file 4: Supplementary File S2). One could argue that the prediction features used in this analysis rely excessively on existing knowledge. Should this be the case, predictions for all the variants in a particular kinase group, protein kinase or PFAM domain would follow the same character, being all either neutral or pathogenic. Most of the 53 protein kinases that harbored variants predicted as disease-associated also presented neutral variation (Fig. 2, panel a). The same is also true for kinase groups and PFAM domains (Fig. 2, panels c, d and e). These results support our selection of features, most importantly, the highly informative accumulative log odds ratio of Gene Ontology terms as a proxy for protein function (Fig. 2, panel f). In spite of being distributed satisfactorily, the results from KinMutRF highlight the functional relevance of previously reported domains such as the protein kinase domain or the PI3K/PI4K and certain taxonomical kinase groups characterised by them, namely Tyr, atypical PI3/PI4 kinase, CAMK and TKL.

## Conclusions

Here we presented a novel method for prioritization of pathogenic variants in the human protein kinase superfamily. KinMutRF implements a random forest classifier that outperforms our previous implementation (KinMut) and other state-of-the-art methods with a similar purpose. Our choice of features and datasets makes the method especially relevant in the context of kinase variantion and their intrinsic role in cancer biology. The family-specific character of the KinMutRF classifier allowed us to introduce features that are unique to the protein kinase family. An analysis of the individual information gain identified these kinase-specific features among the most relevant for a correct classification. Namely, the functional characterization of the kinase according to Gene Ontology terms, the membership to a particular kinase group or the occurrence of the variants at relevant catalytic protein kinase domain arise as important features that are unique to the protein kinase superfamily. This is in full agreement with previous observations and advocates for the urgent development of family-specific classifiers where the abundance of variation data permits.

### Availability of supporting data

KinMutRF is publicly implemented as a component of our pipeline for the identification, annotation and interpretation of the consequences of kinase variants, wKinMut-2 [61]. This resource is freely available at http://kinmut2.bioinfo.cnio.es. The source code, documentation and examples for KinMutRF can be downloaded for local installation from https://github.com/Rbbt-Workflows under a GPV version 3 licence. We are also grateful to the two anonymous reviewers that revised this manuscript for their very relevant comments.

### Consent for publication

Not applicable.

### Ethics approval and consent to participate

Not applicable.

### Availability of data and materials

Training datasets used for 10-fold cross-validation experiment provided as Additional file 2: Supplementary File S1. Predictions for the unclassified variants in Uniprot and the Bruton agammaglobulinemia tyrosine kinase domain are available as Additional file 2: Supplementary Files S1 and Additional file 4: Supplementary Files S2 respectively. The source code of KinMutRF is released under a GPL version 3 license, and can be downloaded from https://github.com/Rbbt-Workflows/KinMut2 whereas a web implementation of KinMutRF is freely available at http://kinmut2.bioinfo.cnio.es.

## Additional files

Additional file 1: Table S1. Description of the characteristics of other state-of-the-art variant pathogenicity prediction methods and classifiers. (DOCX 9 kb) Additional file 2: Supplementary File S1. Training datasets used for 10-fold cross-validation experiment. File formats include weka and plain text. (ZIP 482 kb) Additional file 3: Supplementary File S3. KinMutRF predictions on the human Bruton agammaglobulinemia tyrosine kinase domain. (XLSX 141 kb) Additional file 4: Supplementary File S2. KinMutRF predictions on the unclassified variants in UniProt, including the prediction features that describe each variant. (TXT 155 kb)

## Abbreviations

BTK: Bruton agammaglobulinemia tyrosine kinase
MCC: Mathew’s correlation coefficient

## References

[1] Mardis ER (2011). A decade's perspective on DNA sequencing technology. Nature.

[2] Greenman C, Stephens P, Smith R, Dalgliesh GL, Hunter C, Bignell G, Davies H, Teague J, Butler A, Stevens C, Edkins S, O'Meara S, Vastrik I, Schmidt EE, Avis T, Barthorpe S, Bhamra G, Buck G, Choudhury B, Clements J, Cole J, Dicks E, Forbes S, Gray K, Halliday K, Harrison R, Hills K, Hinton J, Jenkinson A, Jones D (2007). Patterns of somatic mutation in human cancer genomes. Nature.

[3] Sjöblom T, Jones S, Wood LD, Parsons DW, Lin J, Barber TD, Mandelker D, Leary RJ, Ptak J, Silliman N, Szabo S, Buckhaults P, Farrell C, Meeh P, Markowitz SD, Willis J, Dawson D, Willson JKV, Gazdar AF, Hartigan J, Wu L, Liu C, Parmigiani G, Park BH, Bachman KE, Papadopoulos N, Vogelstein B, Kinzler KW, Velculescu VE (2006). The consensus coding sequences of human breast and colorectal cancers. Science.

[4] Wood LD, Parsons DW, Jones S, Lin J, Sjöblom T, Leary RJ, Shen D, Boca SM, Barber T, Ptak J, Silliman N, Szabo S, Dezso Z, Ustyanksky V, Nikolskaya T, Nikolsky Y, Karchin R, Wilson PA, Kaminker JS, Zhang Z, Croshaw R, Willis J, Dawson D, Shipitsin M, Willson JKV, Sukumar S, Polyak K, Park BH, Pethiyagoda CL, Pant PVK (2007). The genomic landscapes of human breast and colorectal cancers. Science.

[5] Creixell P, Schoof EM, Simpson CD, Longden J, Miller CJ, Lou HJ, Perryman L, Cox TR, Zivanovic N, Palmeri A, Wesolowska-Andersen A, Helmer-Citterich M, Ferkinghoff-Borg J, Itamochi H, Bodenmiller B, Erler JT, Turk BE, Linding R (2015). Kinome-wide Decoding of Network-Attacking Mutations Rewiring Cancer Signaling. Cell.

[6] Ritchie GR, Flicek P (2014). Computational approaches to interpreting genomic sequence variation. Genome Med.

[7] Baudot A, Real FX, Izarzugaza JMG, Valencia A (2009). From cancer genomes to cancer models: bridging the gaps. EMBO Rep.

[8] Sahni N, Yi S, Taipale M, Fuxman Bass JI, Coulombe-Huntington J, Yang F, Peng J, Weile J, Karras GI, Wang Y, Kovács IA, Kamburov A, Krykbaeva I, Lam MH, Tucker G, Khurana V, Sharma A, Liu Y-Y, Yachie N, Zhong Q, Shen Y, Palagi A, San-Miguel A, Fan C, Balcha D, Dricot A, Jordan DM, Walsh JM, Shah AA, Yang X (2015). Widespread macromolecular interaction perturbations in human genetic disorders. Cell.

[9] Ng PC, Henikoff S (2001). Predicting deleterious amino acid substitutions. Genome Res.

[10] Reva B, Antipin Y, Sander C. Predicting the functional impact of protein mutations: application to cancer genomics. Nucleic Acids Res. 2011;39:e118–8. 10.1093/nar/gkr407 PMC3177186 21727090

[11] Li B, Krishnan VG, Mort ME, Xin F, Kamati KK, Cooper DN, Mooney SD, Radivojac P (2009). Automated inference of molecular mechanisms of disease from amino acid substitutions. Bioinformatics.

[12] Shihab HA, Gough J, Cooper DN, Stenson PD, Barker GLA, Edwards KJ, Day INM, Gaunt TR (2013). Predicting the functional, molecular, and phenotypic consequences of amino acid substitutions using hidden Markov models. Hum Mutat.

[13] Thomas PD, Kejariwal A, Guo N, Mi H, Campbell MJ, Muruganujan A, Lazareva-Ulitsky B (2006). Applications for protein sequence-function evolution data: mRNA/protein expression analysis and coding SNP scoring tools. Nucleic Acids Res.

[14] Choi Y, Chan AP (2015). PROVEAN web server: a tool to predict the functional effect of amino acid substitutions and indels. Bioinformatics.

[15] Ferrer-Costa C, Gelpí JL, Zamakola L, Parraga I, la Cruz de X, Orozco M (2005). PMUT: a web-based tool for the annotation of pathological mutations on proteins. Bioinformatics.

[16] Bromberg Y, Rost B (2007). SNAP: predict effect of non-synonymous polymorphisms on function. Nucleic Acids Res.

[17] Adzhubei IA, Schmidt S, Peshkin L, Ramensky VE, Gerasimova A, Bork P, Kondrashov AS, Sunyaev SR (2010). A method and server for predicting damaging missense mutations. Nat Methods.

[18] Johansen MB, Izarzugaza JMG, Brunak S, Petersen TN, Gupta R (2013). Prediction of disease causing non-synonymous SNPs by the Artificial Neural Network Predictor NetDiseaseSNP. PLoS ONE.

[19] Karchin R, Diekhans M, Kelly L, Thomas DJ, Pieper U, Eswar N, Haussler D, Sali A (2005). LS-SNP: large-scale annotation of coding non-synonymous SNPs based on multiple information sources. Bioinformatics.

[20] Capriotti E, Calabrese R, Casadio R (2006). Predicting the insurgence of human genetic diseases associated to single point protein mutations with support vector machines and evolutionary information. Bioinformatics.

[21] Schwarz JM, Rödelsperger C, Schuelke M, Seelow D (2010). MutationTaster evaluates disease-causing potential of sequence alterations. Nat Methods.

[22] Carter H, Douville C, Stenson PD, Cooper DN, Karchin R (2013). Identifying Mendelian disease genes with the variant effect scoring tool. BMC Genomics.

[23] Calabrese R, Capriotti E, Fariselli P, Martelli PL, Casadio R (2009). Functional annotations improve the predictive score of human disease-related mutations in proteins. Hum Mutat.

[24] Yue P, Melamud E, Moult J (2006). SNPs3D: candidate gene and SNP selection for association studies. BMC Bioinformatics.

[25] Wainreb G, Ashkenazy H, Bromberg Y, Starovolsky-Shitrit A, Haliloglu T, Ruppin E, Avraham KB, Rost B, Ben-Tal N (2010). MuD: an interactive web server for the prediction of non-neutral substitutions using protein structural data. Nucleic Acids Res.

[26] Kaminker JS, Zhang Y, Watanabe C, Zhang Z (2007). CanPredict: a computational tool for predicting cancer-associated missense mutations. Nucleic Acids Res.

[27] Kircher M, Witten DM, Jain P, O'Roak BJ, Cooper GM, Shendure J (2014). A general framework for estimating the relative pathogenicity of human genetic variants. Nat Genet.

[28] Niroula A, Urolagin S, Vihinen M (2015). PON-P2: prediction method for fast and reliable identification of harmful variants. PLoS ONE.

[29] Bao L, Zhou M, Cui Y (2005). nsSNPAnalyzer: identifying disease-associated nonsynonymous single nucleotide polymorphisms. Nucleic Acids Res.

[30] Capriotti E, Altman RB, Bromberg Y (2013). Collective judgment predicts disease-associated single nucleotide variants. BMC Genomics.

[31] González-Pérez A, López-Bigas N (2011). Improving the assessment of the outcome of nonsynonymous SNVs with a consensus deleteriousness score, Condel. Am J Hum Genet.

[32] Olatubosun A, Väliaho J, Härkönen J, Thusberg J, Vihinen M (2012). PON-P: integrated predictor for pathogenicity of missense variants. Hum Mutat.

[33] Bendl J, Stourac J, Salanda O, Pavelka A, Wieben ED, Zendulka J, Brezovsky J, Damborsky J (2014). PredictSNP: robust and accurate consensus classifier for prediction of disease-related mutations. PLoS Comput Biol.

[34] Wu J, Li Y, Jiang R (2014). Integrating multiple genomic data to predict disease-causing nonsynonymous single nucleotide variants in exome sequencing studies. PLoS Genet.

[35] Mao Y, Chen H, Liang H, Meric-Bernstam F, Mills GB, Chen K (2013). CanDrA: cancer-specific driver missense mutation annotation with optimized features. PLoS ONE.

[36] Carter H, Chen S, Isik L, Tyekucheva S, Velculescu VE, Kinzler KW, Vogelstein B, Karchin R (2009). Cancer-specific high-throughput annotation of somatic mutations: computational prediction of driver missense mutations. Cancer Res.

[37] Izarzugaza JMG, del Pozo A, Vazquez M, Valencia A (2012). Prioritization of pathogenic mutations in the protein kinase superfamily. BMC Genomics.

[38] Izarzugaza JMG, Krallinger M, Valencia A (2012). Interpretation of the consequences of mutations in protein kinases: combined use of bioinformatics and text mining. Front Physiol.

[39] Torkamani A, Schork NJ (2007). Accurate prediction of deleterious protein kinase polymorphisms. Bioinformatics.

[40] Torkamani A, Schork NJ (2008). Prediction of cancer driver mutations in protein kinases. Cancer Res.

[41] Krallinger M, Izarzugaza JMG, Rodriguez-Penagos C, Valencia A (2009). Extraction of human kinase mutations from literature, databases and genotyping studies. BMC Bioinformatics.

[42] Stratton MR, Campbell PJ, Futreal PA (2009). The cancer genome. Nature.

[43] Izarzugaza JMG, Redfern OC, Orengo CA, Valencia A (2009). Cancer-associated mutations are preferentially distributed in protein kinase functional sites. Proteins.

[44] Izarzugaza JMG, Hopcroft LEM, Baresic A, Orengo CA, Martin ACR, Valencia A (2011). Characterization of pathogenic germline mutations in human protein kinases. BMC Bioinformatics.

[45] Izarzugaza JMG, Vazquez M, del Pozo A, Valencia A (2013). wKinMut: an integrated tool for the analysis and interpretation of mutations in human protein kinases. BMC Bioinformatics.

[46] Yip YL, Famiglietti M, Gos A, Duek PD, David FPA, Gateau A, Bairoch A (2008). Annotating single amino acid polymorphisms in the UniProt/Swiss-Prot knowledgebase. Hum Mutat.

[47] Vihinen M (2012). How to evaluate performance of prediction methods? Measures and their interpretation in variation effect analysis. BMC Genomics.

[48] Baldi P, Brunak S, Chauvin Y, Andersen CA, Nielsen H (2000). Assessing the accuracy of prediction algorithms for classification: an overview. Bioinformatics.

[49] Manning G, Plowman GD, Hunter T, Sudarsanam S (2002). Evolution of protein kinase signaling from yeast to man. Trends Biochem Sci.

[50] Manning G, Whyte DB, Martinez R, Hunter T, Sudarsanam S (2002). The protein kinase complement of the human genome. Science.

[51] Ng PC, Henikoff S (2003). SIFT: Predicting amino acid changes that affect protein function. Nucleic Acids Res.

[52] Liu X, Jian X, Boerwinkle E (2013). dbNSFP v2.0: a database of human non-synonymous SNVs and their functional predictions and annotations. Hum Mutat.

[53] UniProt Consortium (2014). Activities at the Universal Protein Resource (UniProt). Nucleic Acids Res.

[54] Dinkel H, Chica C, Via A, Gould CM, Jensen LJ, Gibson TJ, Diella F (2011). Phospho.ELM: a database of phosphorylation sites--update. Nucleic Acids Res 2011.

[55] Lopez G, Valencia A, Tress M (2007). FireDB--a database of functionally important residues from proteins of known structure. Nucleic Acids Res.

[56] Grimm DG, Azencott C-A, Aicheler F, Gieraths U, MacArthur DG, Samocha KE, Cooper DN, Stenson PD, Daly MJ, Smoller JW, Duncan LE, Borgwardt KM (2015). The evaluation of tools used to predict the impact of missense variants is hindered by two types of circularity. Hum Mutat.

[57] Molina-Vila MA, Nabau-Moretó N, Tornador C, Sabnis AJ, Rosell R, Estivill X, Bivona TG, Marino-Buslje C (2014). Activating mutations cluster in the “molecular brake” regions of protein kinases and do not associate with conserved or catalytic residues. Hum Mutat.

[58] Simonetti FL, Tornador C, Nabau-Moretó N, Molina-Vila MA, Marino-Buslje C (2014). Kin-Driver: a database of driver mutations in protein kinases. Database (Oxford).

[59] Väliaho J, Faisal I, Ortutay C, Smith CIE, Vihinen M (2015). Characterization of all possible single-nucleotide change caused amino acid substitutions in the kinase domain of Bruton tyrosine kinase. Hum Mutat.

[60] Vazquez M, Valencia A, Pons T (2015). Structure-PPi: a module for the annotation of cancer-related single-nucleotide variants at protein-protein interfaces. Bioinformatics.

[61] Vazquez M, Pons T, Brunak S, Valencia A, Izarzugaza JMG (2015). wKinMut-2: Identification and Interpretation of Pathogenic Variants in Human Protein Kinases. Hum Mutat.

