# Supplementary material for: KinMutRF: a random forest classifier of sequence variants in the human protein kinase superfamily
Source: BMC Genomics. 2016 Jun 23;17(Suppl 2):396. doi: 10.1186/s12864-016-2723-1 (PMC4928150; doi:10.1186/s12864-016-2723-1)
Supplement: Additional file 1: Table S1. — Description of the characteristics of other state-of-the-art variant pathogenicity prediction methods and classifiers. (DOCX 9 kb) [file 12864_2016_2723_MOESM1_ESM.docx]

| Method | Method Description | DataBase | Features * | Website |
| --- | --- | --- | --- | --- |
| MutPred | Random Forest | HGMD  Swiss-Prot | (i) evolutionary information  (ii) physicochemical properties of the proteins | *http://mutpred.mutdb.org/* |
| SNAP | Neural Networks | PMD  Swiss-Prot  Pfam | (i) evolutionary information  (ii) physicochemical properties of the proteins  (iii) protein domains | [*http://www.bio-sof.com/snap*](http://www.bio-sof.com/snap) |
| PolyPhen2 | Naïve Bayesian Classification | HumDiv (UniProt)  HumVar (UniProt) | (i) evolutionary information  (ii) physicochemical properties of the proteins  (iii) protein domains  (iv) sequence context. | *http://genetics.bwh.harvard.edu/pph2/* |
| nsSNPAnalyzer | Random Forest | Swiss-Prot  ASTRAL | (i) evolutionary information  (ii) physicochemical properties of the proteins | *http://snpanalyzer.uthsc.edu/* |
| SNPs&GO | Support Vector Machine | Swiss-Prot | (i) evolutionary information  (ii) physicochemical properties of the protein  (iv) sequence context. | *http://snps-and-go.biocomp.unibo.it/snps-and-go/* |
| PhD-SNP | Support Vector Machine | Swiss-Prot | (i) evolutionary information  (ii) physicochemical properties of the proteins | *http://snps.biofold.org/phd-snp/phd-snp.html* |
| SIFT | Mathematical Model | Swiss-Prot  Swiss-Prot / TrEMBL  NCBI | (i) evolutionary information | *http://sift.jcvi.org/* |
| Mutation Assessor | Mathematical Model | COSMIC  UniProt  IARC TP53 | (i) evolutionary information | *http://mutationassessor.org/* |
| PON-P2 | Random Forest | VariBench | (i) evolutionary information  (ii) physicochemical properties of the proteins  (iv) sequence context. | *http://structure.bmc.lu.se/PON-P2/* |
| PMUT | Neural Networks | NEMUS  Pfam  Swiss-Prot/trEMBL | (i) evolutionary information  (ii) physicochemical properties of the proteins | *http://mmb2.pcb.ub.es:8080/PMut/* |
| NetDiseaseSNP | Neural Networks | HGMD  UniProt  NCBI non-redundant protein database | (i) evolutionary information  (ii) physicochemical properties of the proteins | *http://www.cbs.dtu.dk/services/NetDiseaseSNP/* |
| LS-SNP | Support Vector Machine | dbSNP (NCBI)  Swiss-Prot/TrEMBL | (i) evolutionary information  (ii) physicochemical properties of the proteins | *http://ls-snp.icm.jhu.edu/ls-snp-pdb/* |
| SNPs3D | Support Vector Machine | NCBI gene database  dbSNP (NCBI)  BIND  HMGD | (i) evolutionary information  (ii) physicochemical properties of the proteins | *http://www.snps3d.org/* |
| FATHMM | Hidden Markov Models | HMGD  UniProt  VariBench  Swiss-Var | (i) evolutionary information | *http://fathmm.biocompute.org.uk/* |
| MutationTaster | Naïve Bayesian Classification | Swiss-Prot/Uniprot  Ensembl  HapMap | (i) evolutionary information  (ii) physicochemical properties of the proteins  (iv) sequence context | *http://www.mutationtaster.org/* |
| VEST | Random Forest | HGMD  Exome Sequencing Project | (i) evolutionary information  (ii) physicochemical properties of the proteins  (iii) protein domains | *http://karchinlab.org/apps/appVest.html* |
| CADD | Support Vector Machine | Ensembl  ENCODE  NIH ClinVar | (i) evolutionary information  (ii) physicochemical properties of the proteins  (iii) protein domains | *http://cadd.gs.washington.edu/* |
| Spring | Statistical Model | OMIM  UniProtKB/Swiss-Prot  dbNSFP  Pfam-A  KEGG | (i) evolutionary information  (ii) physicochemical properties of the proteins  (iii) protein domains | *http://omictools.com/spring-s8720.html* |
| Panther | Mathematical | HMGD  dbSNP (NCBI) | (i) evolutionary information | *http://www.pantherdb.org/tools/csnpScoreForm.jsp* |
| PROVEAN | Mathematical | UniProtKB/Swiss-Prot  HMGD | (i) evolutionary information | *http://provean.jcvi.org/index.php* |
| MuD | Random Forest | UniProtKB/Swiss-Prot  ConSurfDB | (i) evolutionary information  (ii) physicochemical properties of the proteins | *http://mud.tau.ac.il/overview.php* |
| PON-P | Random Forest | dbSNP12 (NCBI)  PhenCode  IDbases | PhD-SNP  SIFT  PolyPhen-2  SNAP  I-Mutant | *http://bioinf.uta.fi/PON-P/.* |
| Condel | Mathematical Model | HumVar (Swiss-Prot)  COSMIC  IARC TP53 | Log R Pfam E-value (Logre)  MAPP  Mutation Assessor  Polyphen2  SIFT | *http://bg.upf.edu/fannsdb/help* |
| PredictSNP | Random Forest | PMD  UniProt | MAPP  nsSNPAnalyzer  Panther  PhD-SNP  Polyphen-1  Polyphen-2  SIFT  SNAP | *http://loschmidt.chemi.muni.cz/predictsnp/* |
| Meta-SNP | Random Forest | Swiss-Var | Panther  PhD-SNP  SIFT  SNAP | *http://snps.biofold.org/meta-snp/* |
| KinMut | Support Vector Machine | UniProt  Phospho ELM  FireDB | (i) evolutionary information  (ii) physicochemical properties of the proteins  (iii) protein domains | *http://kinmut.bioinfo.cnio.es/* |
| Torkami | Support Vector Machines | OMIM  KinMutBase  HMGD  dbSNP125 (NCBI)  PupaSNP | (i) evolutionary information  (ii) physicochemical properties of the proteins  (iii) protein domains  (iv) sequence context |  |
| CanPredict | Random Forest | COSMIC  dbSNP (NCBI) | (i) evolutionary information  (iv) protein domains | *http://research-public.gene.com/Research/genentech/canpredict/* |
| CanDrA | Support Vector Machine | COSMIC (V58)  TCGA  CCLE project. | (i) evolutionary information  (ii) physicochemical properties of the proteins  (iii) protein domains  (iv) sequence context. | *http://bioinformatics.mdanderson.org/main/CanDrA* |
| CHASM | Random Forest | COSMIC | (i) evolutionary information  (ii) physicochemical properties of the proteins  (iii) protein domains  (iv) sequence context. | *http://wiki.chasmsoftware.org/index.php/Main_Page* |

In Blue, Consensus Classifiers

In Pink, cancer specific

In Orange, Kinase Specific

*A Described by : Mao Y, Chen H, Liang H, Meric-Bernstam F, Mills GB, Chen K. CanDrA: Cancer-Specific Driver Missense Mutation Annotation with Optimized Features. *PLoS One*. 2013;8(10):e77945. doi:10.1371/journal.pone.0077945.
